# 2562 Racial/ethnic variation in the relationship between metabolic syndrome components and cardiovascular disease and the role of uric acid among population with metabolic syndrome

**DOI:** 10.1017/cts.2018.180

**Published:** 2018-11-21

**Authors:** Magda Shaheen

**Affiliations:** David Geffen School of Medicine, UCLA

## Abstract

OBJECTIVES/SPECIFIC AIMS: To examine the racial/ethnic variation in the relation between metabolic syndrome (MetS) components and cardiovascular disease (CVD) as well as examine the role of uric acid as a predictor of CVD among population with MetS. METHODS/STUDY POPULATION: We analyzed National Health and Nutrition Examination Surveys data (1999–2010) for adults aged >20 years with MetS. Using the ATP III clinical criteria for diagnosing MetS, subjects were classified as having MetS if they had ≥3 of the following: waist circumference ≥40 inches for men or ≥35 inches for women, triglyceride ≥150 mg/dL, HDL-C for men ≤40 mg/dL; women ≤50 mg/dL, pre-hypertension, or fasting glucose ≥110 mg/dL. We used multiple logistic regression in STATA 14 survey module to examine the relation between MetS components and CVD adjusting for age, gender, race/ethnicity, education, smoking, alcohol, albuminuria, glomerular filtration rate, C-reactive protein, uric acid and white blood count. To assess the racial/ethnic variation, we examined the same model in each race/ethnic group. RESULTS/ANTICIPATED RESULTS: Of the 3212 subjects, 78% were Whites, 10% were Blacks, and 15% had CVD. MetS components, CVD, and uric acid varied significantly by race/ethnicity (*p*<0.05). In the multivariate model, HDL-C level [odds ratio (OR)=1.5; 95% confidence interval (CI)=1.1–2.0], triglyceride level (OR=2.0; CI=1.4–2.9), and elevated uric acid (OR=1.4; CI=1.1–1.9) were independently related to CVD (*p*<0.05). While CVD was independently related to HDL-C, triglyceride, and elevated uric acid in Whites (*p*<0.05), it was associated with pre-hypertension and triglyceride in Blacks (*p*<0.05) and no predictors in Hispanics (*p*>0.05). DISCUSSION/SIGNIFICANCE OF IMPACT: Elevated uric acid, HDL-C, and triglyceride levels are significant independent predictors of CVD among population with MetS. These predictors varied by race/ethnicity. Health care providers should be vigilant in the management of MetS components and control of uric acid level in each racial/ethnic group to prevent the CVD risk among the population with MetS.

